# Ferulic Acid Attenuates Hypoxia/Reoxygenation Injury by Suppressing Mitophagy Through the PINK1/Parkin Signaling Pathway in H9c2 Cells

**DOI:** 10.3389/fphar.2020.00103

**Published:** 2020-02-25

**Authors:** Chenxi Luo, Yehao Zhang, Hao Guo, Xiao Han, Junguo Ren, Jianxun Liu

**Affiliations:** ^1^ Graduate School, Beijing University of Chinese Medicine, Beijing, China; ^2^ Beijing Key Laboratory of Pharmacology of Chinese Materia Region, Institute of Basic Medical Sciences, Xiyuan Hospital, China Academy of Chinese Medical Sciences, Beijing, China

**Keywords:** ferulic acid, hypoxia/reoxygenation, mitochondrial dysfunction, mitophagy, PINK1/Parkin

## Abstract

Ferulic acid protects against cardiac injury by scavenging free radicals. However, the role of mitophagy in ferulic acid-induced cardioprotection remains obscure. In the present study, H9c2 cells were exposed to hypoxia/reoxygenation and ferulic acid treatment during hypoxia. We illustrated the impact of ferulic acid on oxidative damage in H9c2 cells. Our results showed that ferulic acid significantly attenuated apoptosis induced by hypoxia/reoxygenation injury and reduced mitochondrial dysfunction, evidenced by a decline in the overproduction of reactive oxygen species and ATP depletion and recovery of the membrane potential. We also found that mitophagy, a selective form of autophagy, was excessively activated in H9c2 cells subjected to hypoxia/reoxygenation. Ferulic acid reduced the binding of mitochondria to lysosomes, down-regulated the PINK1/Parkin pathway, and was accompanied by increased p62 and decreased LC3-II/LC3-I levels. Ferulic acid also antagonistically reduced the activation of mitophagy by rapamycin. These findings suggest that ferulic acid may protect H9c2 cells against ischemia/reperfusion injury by suppressing PINK1/Parkin-dependent mitophagy. Accordingly, our findings may provide a potential target and powerful reference for ferulic acid in clinical prevention and treatment of hypoxia/reoxygenation injury.

## Introduction

Myocardial ischemia/reperfusion (I/R) injury is a complex process that causes damage to proteins, DNA, and plasma membranes, thereby promoting cell death and diminished cardiac output (Hausenloy and Yellon, 2013; Go et al., 2014). Mitochondrial dysfunction is a basic pathophysiological factor of I/R injury (Walters et al., 2012). Damaged mitochondria produce less ATP, generate greater amounts of reactive oxygen species (ROS), and activate mitochondria‐dependent apoptosis (Ong and Gustafsson, 2012; Chen and Zweier, 2014). Cardiomyocytes, which depend heavily on energy generation by mitochondria, are more sensitive to mitochondrial dysfunction (Kasahara and Scorrano, 2014). Thus, preserving healthy mitochondria is imperative to cell survival in cardiomyocytes.

Autophagy is activated by energy consumption, oxidative stress, total protein, and damaged organelles (Tannous et al., 2008; Kim et al., 2013). It is a developmentally preserved process through which cytoplasmic components and organelles are digested and reused in lysosomes (Kroemer et al., 2010). Despite numerous evaluations illustrating the benefits of autophagy, excessive autophagy can induce cell death in myocardial I/R injury (Hamacher-Brady et al., 2006; Matsui et al., 2007). Mitochondrial autophagy (referred to as mitophagy), a selective form of autophagy, degrades dysfunctional mitochondria and regulates the mitochondrial population (Ashrafi and Schwarz, 2013; Hammerling and Gustafsson, 2014). Hypoxemia, starvation, and ROS can trigger mitophagy, which is associated with several forms of neurodegeneration and cardiovascular diseases (Youle and Narendra, 2011; Dorn and Kitsis, 2015). Recent studies suggest that PINK1 (phosphatase and tensin homolog-induced putative kinase 1) and the Parkin pathway play central roles in regulating mitophagy and mitochondrial quality control (Springer and Kahle, 2011). Furthermore, PINK1/Parkin-mediated mitophagy reportedly prevents cellular damage (Huang et al., 2011). Accordingly, mitophagy may have restorative potential for mitochondria-associated conditions such as I/R injury.

Ferulic acid (4-hydroxy-3-methoxy cinnamic acid, FA) is a kind of phenolic acid widely distributed in various plants (Pan et al., 2016). It is also an active ingredient of traditional Chinese medicines such as *Angelica sinensis* (Oliv.) Diels, *Ligusticum chuanxiong* Hort. and *Cimicifuga foetida* L. It has various biological activities, such as the scavenging of free radicals, anti-oxidation, anti-platelet aggregation, neuroprotection, and enhancement of immune function (Barone et al., 2009; Mancuso and Santangelo, 2014). Current research about the antioxidant mechanisms of FA have been focused on the scavenging of free radicals and inhibition of myocardial apoptosis (Alam et al., 2013). The potential role of FA in mitophagy has not yet been reported.

Therefore, we investigated the defensive impact of FA in cardiomyocytes against hypoxia/reoxygenation (H/R)‐induced mitophagy, with a focus on the PINK1/Parkin pathway.

## Material and methods

### Cell Culture and Treatment

Hypoxia/reoxygenation experiments were performed based on a previous method (Tang et al., 2013; Zhang et al., 2018). The H9c2 cells were cultured in Dulbecco′s Modified Eagle′s medium (DMEM) (Hyclone, China) supplemented with 10% (v/v) fetal bovine serum (FBS; Gibco, USA) and 1% penicillin/streptomycin (Sigma, St. Louis, MO) at 37°C in a culture box, with an atmosphere of 95% air and 5% CO_2_. Then, the cells were placed in a pre-mixed gas (94% N_2_, 5% CO_2_, 1% O_2_) culture box and cultured in deoxygenated DMEM without glucose and FBS for 2 hours. After 2 h, normal DMEM with 10% FBS serum was given, and cells were transferred to an atmosphere incubator for 2 h. The control group cells were cultured with normal DMEM. During hypoxia stimulation, cells were treated for 2 h with ferulic acid (FA, Beijing Laiyao Biotechnology Co., Ltd, China), with or without rapamycin (autophagy activator, Rapa, 100 nM, Beijing Laiyao Biotechnology Co., Ltd, China) (Hausenloy and Yellon, 2013; Sun et al., 2018).

### Cell Viability Assay

After the hypoxia/reoxygenation stimulation, H9c2 cells were treated with the CCK-8 assay (10 µL/well, Dojindo, Kumamoto, Japan) for an additional 2 h. The absorbance was recorded at 450 nm using a microplate absorbance reader (BioTek, USA).

### TUNEL Staining

After the indicated treatments, cells were fixed with 4% paraformaldehyde solution for 25 min at room temperature, and then washed with phosphate-buffered saline (PBS, Gibco, USA). The cells were then permeabilized with 0.2% Triton X-100 for 5 min. After washing with PBS, apoptosis was detected using a terminal deoxynucleotidyl transferase-mediated-dUTP nick end-labeling (TUNEL) kit (Roche, Indianapolis, IN, USA) according to the supplier′s instructions. DAPI counterstaining was performed to stain the nuclei. Cells were observed and photographed using a fluorescence microscope (Olympus, Tokyo, Japan). Quantitative analysis was presented as average counts of TUNEL-positive cells per field counted in five random fields with 350-400 total cells in each field and three independent experiments for each group.

### ADP/ATP Ratio Measurements

The ATP/ADP level in cells was measured using a bioluminescent detection kit (ADP/ATP Ratio Assay Kit, Abcam) according to the manufacturer′s instructions. After the indicated treatments, the bioluminescent intensities were measured on a multi-mode microplate reader (Synergy H1 Hybrid, BioTek).

### Mitochondrial Membrane Potentials Assay

The mitochondrial membrane potential was assessed using 5,5′,6,6′ -tetrachloro-1,1′,3,3′ -tetraethyl-benzimidazolecarbocyanide iodine (JC-1, Sigma, St. Louis, MO, USA). The H9c2 cells were incubated with JC-1 staining solution (10 µg/mL) for 20 min at 37°C in the dark and rinsed twice with PBS. The JC-1 fluorescence was measured by a fluorescence microplate reader at an excitation of 485 nm and emission of 535 nm (monomer form of JC-1, green), and excitation of 550 nm and emission of 600 nm (aggregate form of JC-1, red). The ratio of green and red fluorescence intensities reflected the changes in mitochondrial membrane potential.

### Determination of ROS

After the indicated treatments, cells were collected, washed once with 1× wash buffer and stained with 20 µM 2′,7′-dichlorofluorescin diacetate (DCFDA) (Abcam, Cambridge, UK) for 30 min in the dark at 37°C, and then analyzed by flow cytometry (FACS Calibur, Becton Dickinson Company, USA) at 485 nm excitation and 535 nm emission.

### Detection of Mitophagy

Mitophagy was determined by the detection of lysosomes. The H9c2 cells were seeded at a density of 1 × 10^5^ in 35-mm glass bottom Petri dishes. The cells were then incubated for 24 h. After the respective treatments, cells were harvested and washed twice. LysoTracker Red (Thermo Fisher Scientific, MA, USA) was then added to the growth medium to achieve a final concentration of 50 nM, and the cells were further incubated for 20 min. MitoTracker Green (Thermo Fisher Scientific, MA, USA) was added to achieve a final concentration of 50 nM, and the cells were again incubated for 15 min. Co-loaded cells were then washed and loaded with phenol red-free DMEM and immediately analyzed by confocal microscopy (Olympus FV1200, Tokyo, Japan). The green fluorescence of MitoTracker Green (excitation of 490 nm and emission of 516 nm) and the red fluorescence of LysoTracker Red (excitation of 647 nm and emission of 668 nm) were measured immediately. At least 50 cells from three independent experiments for each group were included; the counts were averaged.

### Immunofluorescence Analysis

The H9c2 cells were cultured on a glass-bottom dish. After the respective treatments, cells were fixed with 100% methanol for 5 min at room temperature and then were blocked in 1% bovine serum albumin (BSA)/10% normal goat serum/0.3 M glycine in 0.1% PBS-Tween for 1 h at room temperature. The cells were then incubated with rabbit anti-PINK1 (1 µg/mL) and mouse anti-HSP60 (1 µg/mL) overnight at 4°C. The secondary antibodies, Alexa Fluor^®^ 594 goat anti-rabbit IgG (1:200) and Alexa Fluor^®^ 488 goat anti-mouse IgG (1:1000) were then added, and the cells were incubated for 1 h at 37°C. Furthermore, 4′,6-diamidino-2-phenylindole (DAPI) counterstaining wasperformed to stain the nuclei. Co-loaded cell images were obtained using a confocal laser microscope system (FV1000, Olympus, Tokyo, Japan).

Similarly, after the respective treatments, cells were fixed with 4% paraformaldehyde and permeabilized with 0.1% Triton X-100. The cells were then incubated with rabbit anti-Parkin (1:100) and mouse anti-HSP60 (1 µg/mL) overnight at 4°C. The secondary antibodies, Alexa Fluor^®^ 488 goat anti-rabbit IgG (1:200) and goat anti-mouse IgG (1:1000), were added and the cells were further incubated for 1 h at 37°C. Counterstaining using DAPI was performed, and images were obtained as described above. All antibodies were purchased from Abcam.

### Western Blot Analysis

Proteins were separated using 10% SDS-PAGE gel wells and electrotransferred onto polyvinylidene difluoride (PVDF) membranes. The TBST buffer was added and 5% BSA was used to block nonspecific sites for 60 min. After blocking, blots were probed overnight at 4°C with primary antibodies against SQSTM1/p62 (1:1000, CST, USA); LC3B (1:1000, ABclonal, USA); PINK1 (1:2000, Abcam, USA); Parkin (1:1000, Abcam, USA); Caspase3 (1:1000, Abcam, USA); and β-actin (1:2000, Abcam, China), followed by horseradish peroxidase-conjugated secondary antibody for 90 min at 37°C. The proteins were then visualized by chemiluminescence and exposure to X-ray film (Bio-Rad).

### Statistical Analysis

Data management and analysis were performed using the GraphPad Prism software (San Diego, CA). All data were reported as mean ± SEM. Each experiment was repeated three times. Univariate analysis of variance (ANOVA) was used for multiple comparisons. The Student′s *t*-test was conducted to analyze intergroup comparisons. P < 0.05 were reported as statistically significant.

## Results

### FA Attenuates H/R Injury in H9c2 Cells

**Figure 1:**
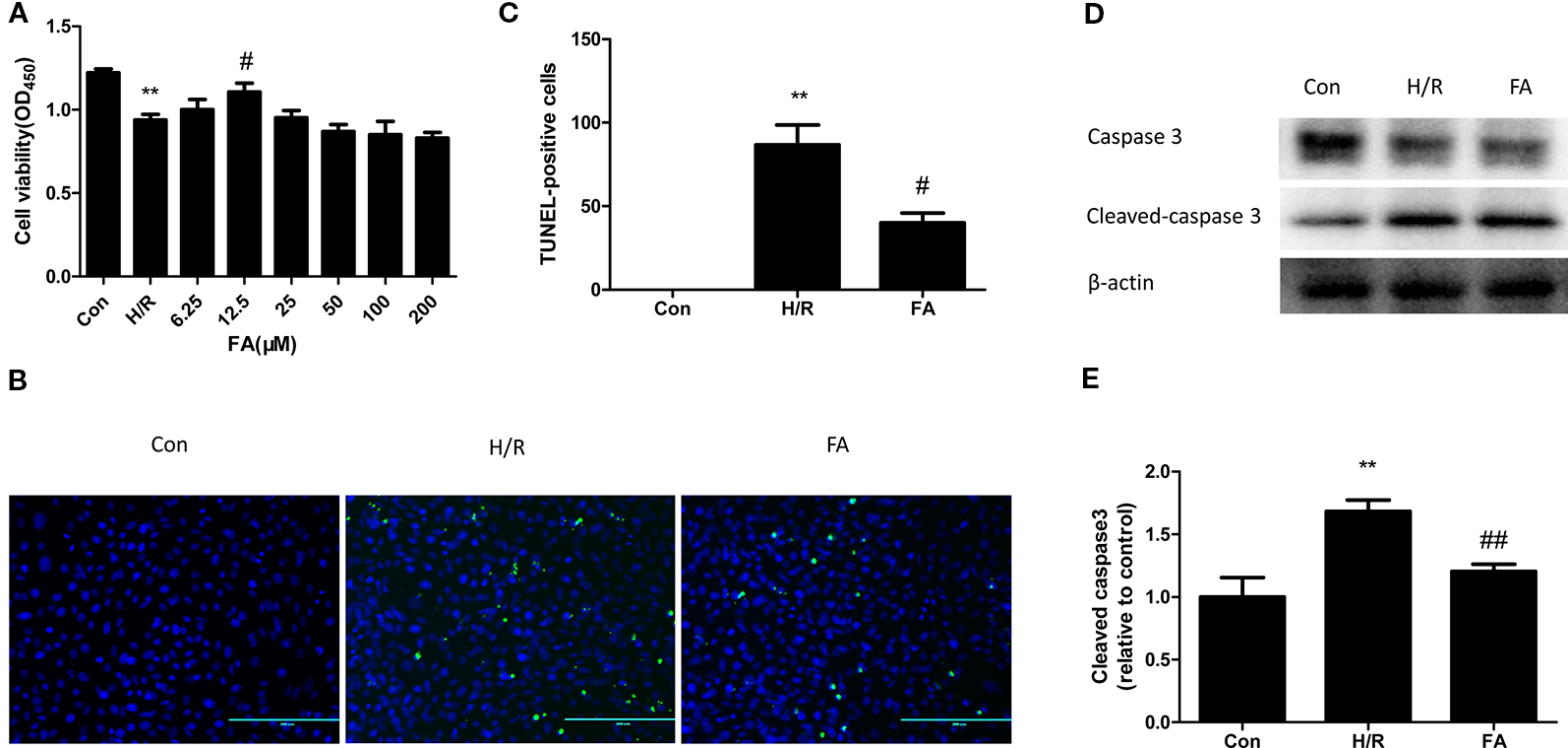
Ferulic acid (FA) attenuated hypoxia/reoxygenation (H/R) injury in H9c2 Cells. **(A)** 12.5 μM FA increased cell viability. **(B**, **C)** FA inhibited the apoptosis levels tested by the TUNEL assay. **(D**, **E)** FA increased the level of cleaved caspase 3. Data were expressed as mean ± SEM (n = 6). **P < 0.01 versus control group; ^#^P < 0.05 and ^##^P < 0.01 versus H/R groups.

The H9c2 cells were subjected to 2 h of hypoxia, followed by 2 h of reoxygenation. The CCK8 assay indicated that H/R treatment significantly reduces myocardial cell viability (P < 0.01, versus the control). FA (25~400 µM) displayed dose-dependent toxicity to H9C2 cells (**Figure S1**), yet the lower concentrations of FA (6.25~200 µM) reduced the damage caused by H/R injury. H9c2 cells were hypoxia for 2 h under different concentrations of FA (6.25, 12.5, 25, 50, 100, and 200 µM). The results showed that FA treatment attenuated the reduction in cell viability caused by H/R injury and reached the highest effective concentration at 12.5 µM (P < 0.05, versus H/R) (**Figure 1A**). Therefore, 12.5 µM FA was administered in the following tests.

Cardiomyocytes are sensitive to ischemia/reperfusion injury and undergo apoptosis due to an insufficient oxygen supply. We conducted the TUNEL assay to analyze the effect of FA on apoptosis during H/R treatment. Compared to the control group, the cells subjected to H/R injury showed a significant increase in apoptotic rate; however, this increase was attenuated by FA (**Figures 1B–C**). To further confirm the results, we used western blot analysis to detect the expression of cleaved caspase3, a key downstream effector protein of apoptosis. The expression of cleaved caspase3 in H9c2 cells was increased during H/R injury, and FA effectively reduced this expression (**Figures 1D–E**). The entire image of capase3 and cleaved caspase3 was shown in **Figure S2**. These data indicated that FA treatment could significantly increase cell viability and reduce apoptosis to attenuate H/R injury in H9c2 cells.

### FA Protected Mitochondrial Function Against H/R Injury

**Figure 2:**
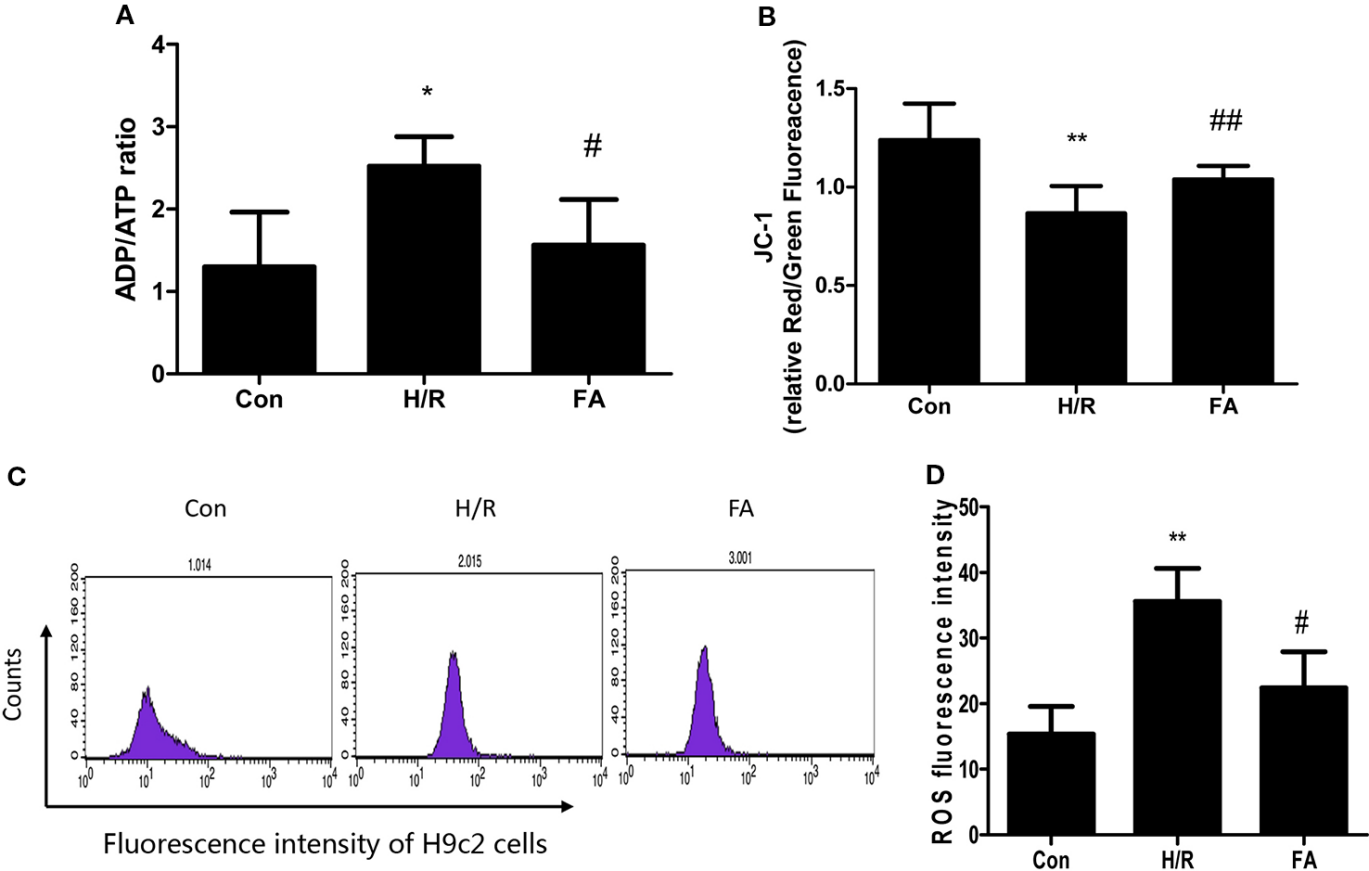
Ferulic acid (FA) alleviated mitochondrial dysfunction following hypoxia/reoxygenation (H/R). **(A)** ADP/ATP ratio. **(B)** JC-1 staining. The ratio of Red/Green fluorescence reflected change in the mitochondrial membrane potential. **(C**, **D)** Cells were analyzed by flow cytometry after being stained with DCFDA to detect ROS. Data were expressed as mean ± SEM (n = 3). *P < 0.05 and **P < 0.01 versus the control group. ^#^P < 0.05 and ^##^P < 0.01 versus the H/R group.

Mitochondria are critical organelles associated with ATP production for cellular metabolism. Therefore, the level of ADP/ATP conversion is one marker of mitochondrial energy metabolism. The ADP/ATP ratio of cardiomyocytes was detected using an ATP bioluminescent kit. Our results showed that H/R injury led to a significant decline in the level of ATP production and FA treatment fully restored energy production in the H9c2 cells (**Figure 2A**).

Mitochondrial function was also assessed by examining changes in the membrane potential. When the mitochondrial membrane potential is normal in H9c2 cells, JC-1 enters the mitochondria through the polarity of the mitochondrial membrane and forms a polymer that emits red fluorescence due to an increase in concentration. However, in damaged cells, the mitochondrial transmembrane is depolarized, and JC-1 is released from the mitochondria, thereby reducing its concentration, and is reversed to a monomeric form that emits green fluorescence. The relative proportion of commonly used green-red fluorescence is a measure of the ratio of changes in the mitochondrial membrane potential. Our results showed that mitochondrial depolarization was aggravated in H/R injury. Nevertheless, FA relieved the decline in membrane potential, which appeared as a significant increase in the Red/Green ratio compared to that in H/R group (**Figure 2B**).

Significant ROS production results in mitochondrial dysfunction. As shown in **Figures 2C, D**, ROS levels were significantly elevated following H/R injury, and treatment with FA significantly reduced ROS generation compared with the untreated cells. Overall, these data indicate that FA treatment could alleviate mitochondrial dysfunction following H/R.

### FA Inhibits Mitophagy in H/R Injury

**Figure 3:**
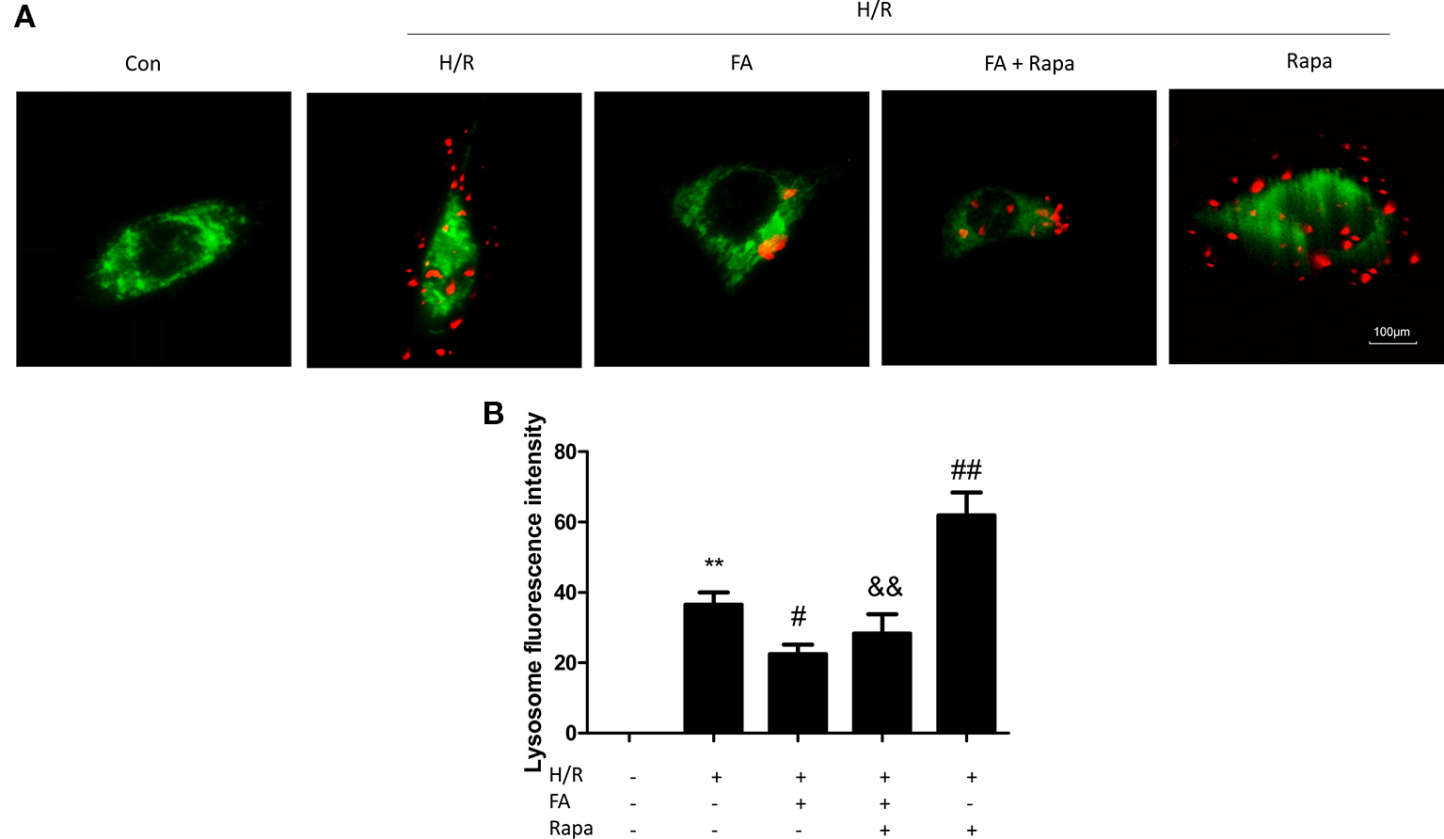
Ferulic acid (FA) inhibited mitophagy. **(A)** Mitophagy was detected by using MitoTracker Green and LysoTracker Red staining. **(B)** Quantitative analysis of lysosome fluorescence intensity. At least 50 cells from three independent experiments for each group were included; the counts were averaged. Scale bar: 100 µm. Data were expressed as the mean ± SEM (n = 3). **P < 0.01 versus the control group. ^#^P < 0.05 and ^##^P < 0.01 versus the hypoxia/reoxygenation (H/R) group. ^&&^P < 0.01 versus the rapamycin group.

Excessive autophagy induces cell death in myocardial I/R injury (Hamacher-Brady et al., 2006; Matsui et al., 2007). As FA protected mitochondrial function and attenuated H/R injury, we hypothesized that FA may confer mitochondrial protection by inhibiting mitophagy. Thus, rapamycin (Rapa, an autophagy activator) was co-administered with FA at the onset of hypoxia. We used MitoTracker Green and LysoTracker Red co-staining in H9c2 cells and then performed confocal microscopy analysis of co-localization of the mitochondria and lysosomes. Our results showed that H/R injury significantly activated mitophagy, and FA treatment effectively inhibited excessive mitophagy, as indicated by the fluorescence intensity of the red LysoTracker. Furthermore, rapamycin significantly promoted the increased number of lysosomes after H/R, and FA alleviated this increase (**Figure 3**).

### FA Inhibits Mitophagy that was Dependent on PINK1/Parkin Signaling

**Figure 4:**
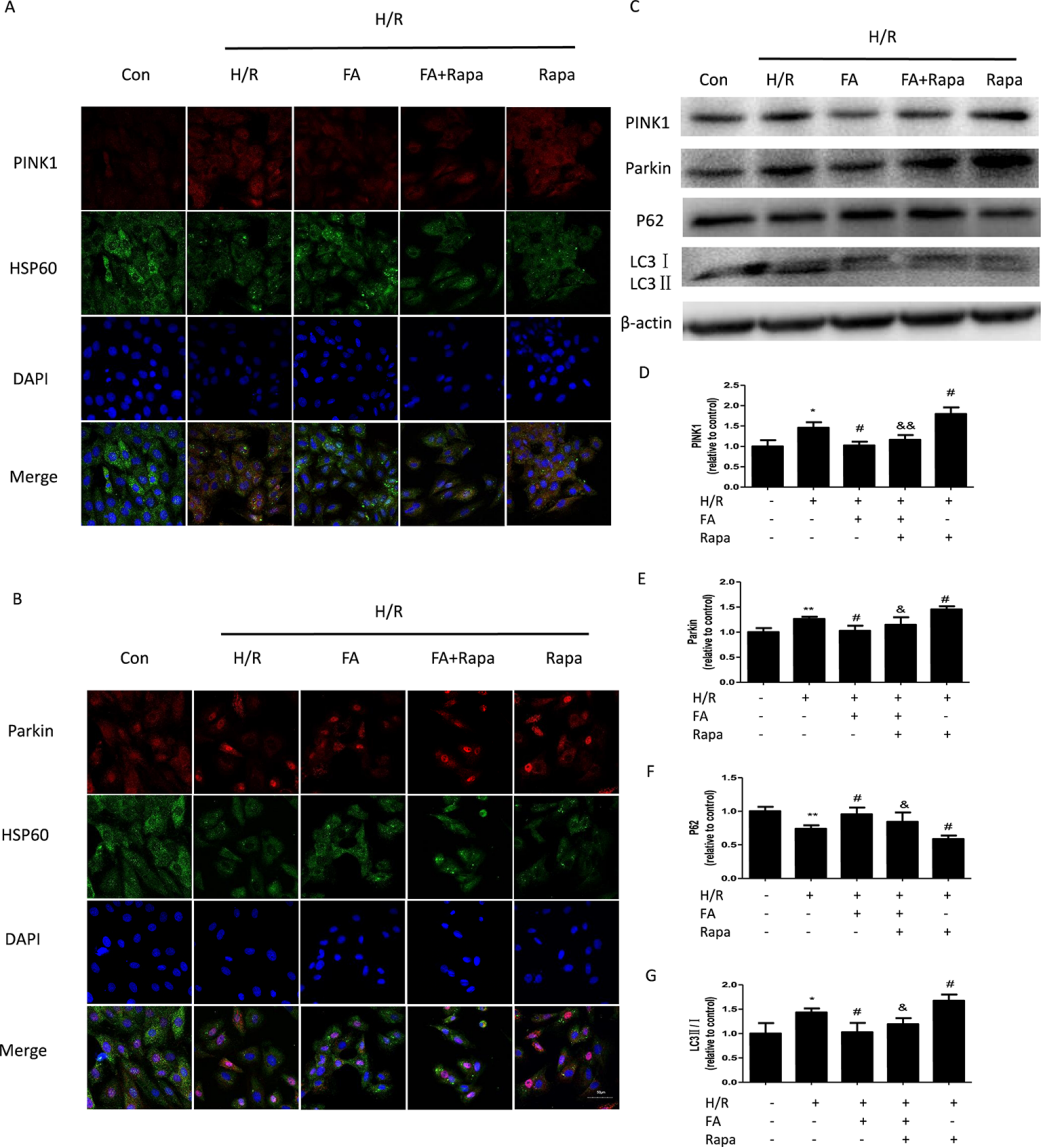
Ferulic acid (FA) inhibited mitophagy that was dependent on PINK1/Parkin. **(A**, **B)** Co-immunofluorescence staining for PINK1/Parkin and HSP60-labeled mitochondria. Scale bar = 50 µm. **(C–G)** Expressions of mitophagy markers, including PINK1, Parkin, LC3, and P62. Data were expressed as the mean ± SEM (n = 3). *P < 0.05 and **P < 0.01 versus the control group. ^#^P < 0.05 versus the H/R group. ^&^P < 0.05 and ^&&^P < 0.01 versus the rapamycin group.

Increasing evidence suggests that the PINK1/Parkin pathway is one of the most important ways by which mitophagy can be mediated (Mokranjac and Neupert, 2007; Matsuda et al., 2010; Moyzis et al., 2015). Therefore, we further investigated whether the inhibition of excessive mitophagy by FA is mediated through the PINK1/Parkin pathway. Autophagy is occurring accompanied with LC3-II (autophagosome membrane type) conversion from LC3-I (cytosolic LC3) and p62 degradation (Tanida et al., 2008). Western blotting analysis showed that compared with control cells, the H/R injury group showed a notable increase in the expression levels of PINK1 and Parkin, which occurred concurrently with the change in the levels of autophagy markers (increases in LC3-II/LC3-I and decline in p62 levels). The FA treatment significantly suppressed the activation of mitophagy in H9c2 cells, as evidenced by the decline in the expression levels of PINK1 and Parkin, with the reduction of LC3-II/LC3-I and accumulation of p62. Likewise, FA antagonistically reduced the activation of rapamycin (**Figures 4C–G**). In addition, co-immunofluorescence staining also demonstrated that the PINK1 and Parkin levels in H9c2 cells were markedly increased after H/R injury but were diminished by FA treatment. Thus, FA partially blocked the activation effects of rapamycin (**Figures 4A, B**).

## Discussion

To our knowledge, this study is the first to establish that FA exerts protective effects against H/R-induced injury in H9c2 cells by suppressing mitophagy. Moreover, FA may offer protection by alleviating H/R-induced apoptosis *via* the down-regulation of PINK1/Parkin-dependent mitophagy and reduction of mitochondrial dysfunction. Overall, our findings showed the pivotal protective effects of FA in H9c2 cells subjected to H/R injury, which were achieved by regulating mitophagy.

Mitochondria perform a double role in the life and death of cardiomyocytes (Bourke et al., 2013). Normal mitochondria produce large quantities of ATP, which drives virtually all biological processes; however, damaged mitochondria produce pathological ROS. Excessive ROS generation attacks the bilayer lipid membrane of cells and subcellular organelles, decreases the mitochondrial membrane potential, and causes mitochondrial dysfunction (Cribbs and Strack, 2007; Detmer and Chan, 2007). Therefore, reducing the number of damaged mitochondria and ensuring mitochondrial quality is an important strategy against cell damage.

Autophagy is the only mechanism by which entire organelles are engulfed and recycled through the lysosomal pathway (Liu et al., 2013). Mitophagy refers to a specific autophagic phenomenon, in which cells are selectively cleared of damaged mitochondria through autophagy (Yang and Klionsky, 2010; Fimia et al., 2013). In general, normal mitophagy enables cells to degrade and remove damaged or dysfunctional mitochondria to maintain a balance of intracellular mitochondrial mass and quantity, and protect cell function (Hamacher-Brady et al., 2007; Kubli et al., 2013). Reducing mitophagy can reportedly lead to inflammation and cell death, which can lead to degenerative diseases (de Vries and Przedborski, 2013). In contrast, studies have shown that excessive mitophagy promotes the loss of cardiomyocytes (Lu et al., 2009; Ji et al., 2016). Recent reports showed that under acute mitochondrial stress, the cell triggers program that result in the elimination of the defective mitochondria, first by promoting mitophagy, then by ensuring that the capacity for autophagosome degradation is increased by building more lysosomes (Deus et al., 2020). Our results suggested that H/R stimulation leads to defective mitophagy concurrently with cell injury and death, evidenced as reduced cell viability, increased expression of cleaved caspase 3, and apoptosis. Moreover, the binding of mitochondria and lysosomes was increased. These results showed that mitophagy was activated in H9c2 cells subjected to H/R. Thus, it was conceivable that inhibiting mitophagy might be beneficial to cardiomyocytes and represent a vital target for I/R injury.

Many studies have shown that FA can improve the clinical symptoms of patients with coronary heart disease and angina. It can also eliminate free radicals and reduce cardiomyocyte apoptosis (Alam et al., 2013). Moreover, new evidence shows that FA can alleviate cardiac injury caused by ischemia/reperfusion. Nevertheless, the exact mechanism of the protective effects of FA on H/R-induced myocardial injury is unclear. The present study demonstrated that FA can reserve mitochondrial function by decreasing ROS overproduction and ATP depletion and recovering the membrane potential. In addition, FA relieved the mitochondrial-based cell injury by inhibiting apoptosis. Moreover, FA administration during hypoxia reduced the binding of mitochondria and lysosomes in H/R cells and inhibited the process of mitophagy, which was activated by the autophagy activator, rapamycin. These findings suggested that mitophagy is an autophagy-related process.

Currently, PINK1/Parkin-directed mitophagy provided a mechanistic link between mitochondrial damage and autophagic clearance (Geisler et al., 2010). We further explored the signaling pathway mediated by FA in H9c2 cells. Upon mitochondrial damage, PINK1 aggregates abundantly on the outer membrane of the mitochondria and recruits Parkin from the cytoplasm to the mitochondria. Once recruited, Parkin ubiquitinates various substrates to induce and promote the autophagic removal of damaged mitochondria (Geisler et al., 2010). Studies have shown that the PINK1/Parkin pathway may be excessively activated in response to I/R injury to induce myocardial cell death (Wang et al., 2012; Ji et al., 2016). Consistent with these findings, we observed that in cultured H9c2 cells, H/R induced significant mitophagy, which was associated with activation of the PINK1/Parkin-mediated mitophagy signaling pathway. Furthermore, FA down-regulated the PINK1/Parkin pathway and suppressed subsequent mitophagy by reducing LC3-II/LC3-I levels and depleting P62 and antagonistically reducing the activation of mitophagy by rapamycin.

## Conclusion

Generally, our findings offered compelling evidence that FA inhibited excessive mitophagy *via* the PINK1/Parkin pathway, thereby restoring mitochondrial function to counteract H/R-induced apoptosis and increase cardiomyocyte survival. This study might facilitate the development of a new application for FA in the prevention and treatment of ischemic heart disease.

## Data Availability Statement

All datasets generated for this study are included in the article/**Supplementary Material**.

## Author Contributions

CL and YZ performed the research study. CL, YZ, and JL designed the research study. CL wrote the first draft of the manuscript. HG, XH, and JR contributed to the revision of the manuscript. All the authors reviewed and approved the submitted version.

## Funding

This study was supported by grants from the National Basic Research Program (973 program) (China) (No.2015CB554405).

## Conflict of Interest

The authors declare that the research was conducted in the absence of any commercial or financial relationships that could be construed as a potential conflict of interest.

## Abbreviations

FBS, fetal bovine serum; CCK8, cell counting kit-8; HSP60, Heat Shock Protein 60; SDS-PAGE, Sodium dodecyl sulfate-Polyacrylamide gel electrophoresis; TBST, Tris Buffered saline Tween; SQSTM1, Sequestosome 1; LC3B, Light Chain 3B.
