# Supplementary material for: Ferulic Acid Attenuates Hypoxia/Reoxygenation Injury by Suppressing Mitophagy Through the PINK1/Parkin Signaling Pathway in H9c2 Cells
Source: Front Pharmacol. 2020 Feb 25;11:103. doi: 10.3389/fphar.2020.00103 (PMC7052384; doi:10.3389/fphar.2020.00103)
Supplement: Supplementary file 1 [file DataSheet_1.docx]

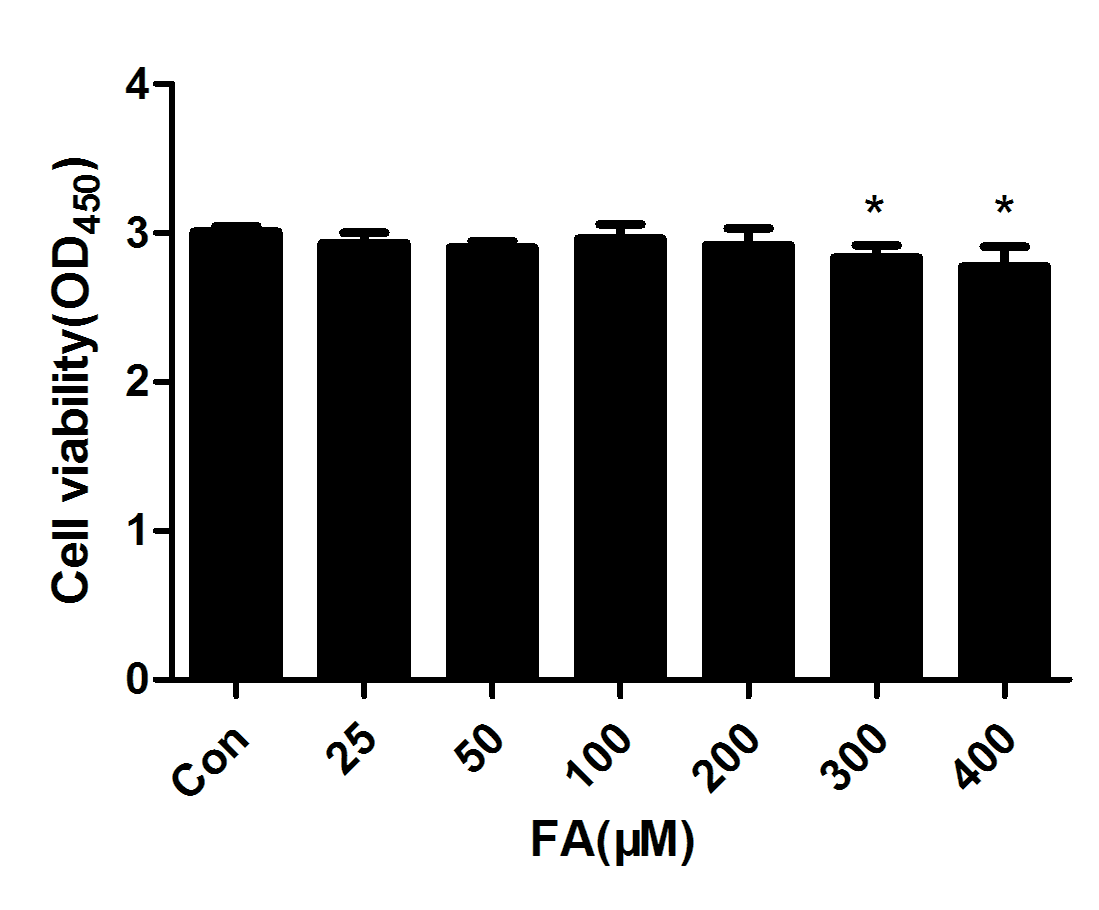


**Figure S1.** Cytotoxicity test of Ferulic acid **(**FA) in H9c2 cells.

H9c2 cells were treated with different dosages of FA for 24h, then cell viability were monitored by the CCK-8 assay. Data are expressed as mean ± SEM (n = 6). **P* < 0.05 *vs.* the control group.


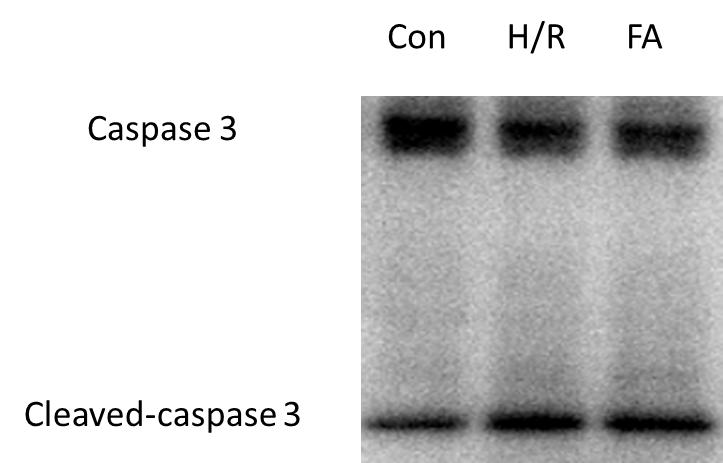


**Figure S2.** The entire get of caspase3 and cleaved-caspase3.
